# Supplementary material for: Study on NGF and VEGF during the Equine Perinatal Period—Part 2: Foals Affected by Neonatal Encephalopathy
Source: Vet Sci. 2022 Aug 26;9(9):459. doi: 10.3390/vetsci9090459 (PMC9503474; doi:10.3390/vetsci9090459)
Supplement: Supplementary file 1 [file vetsci-09-00459-s001.zip › vetsci-1843295-supplementary.pdf]

Table S1. Foals complete blood cell counts, serum biochemistry, electrolyte concentrations, rapid determinations and arterial blood gas analysis at birth (group NE) or at admission (group exNE). Data are expressed as mean ±standard deviation (min-max). For normal values refer to [34], [35], [36], [37], and [38].

[34] Harvey, J.W. Normal hematological values. In *Equine Clinical Neonatology*; Koterba, A.M., Drummond, W.H., Kosch, P.C., Eds.; Lea and Febiger: Philadelphia, 1990; pp. 561-570.

[35] Bauer, J.E.; Harvey, J.W.; Asquith, R.L.; McNulty, P.K.; Kivipelto, J.A.N. Clinical chemistry reference values of foals during the first year of life. *Equine Vet J* **1984**, *16*, 361-363.

[36] Stoneham, S.J.; Palmer, L.; Cash, R.; Rossdale, P.D. Measurement of serum amyloid A in the neonatal foal using a latex agglutination immunoturbidimetric assay: determination of the normal range, variation with age and response to disease. *Equine Vet J* **2001**, *33*, 599-603.

[37] Pirrone, A.; Mariella, J.; Gentilini, F.; Castagnetti, C. Amniotic fluid and blood lactate concentrations in mares and foals in the early postpartum period. *Theriogenology* **2012**, *78*, 1182-1189.

[38] Aguilera-Tejero, E.; Estepa, J.C.; Lopez, I.; Mayer-Valor, R.; Rodriguez, M. Arterial blood gases and acid-base balance in healthy young and aged horses. *Equine Vet J* **1998**, *30*, 352-354.

**Haematology**									
Haemoglobin g/dL	Haematocrit %	Erythrocytes $10^6/\mu L$	Platelets $10^3/\mu L$	Leucocytes $10^3/\mu L$	Lymphocytes $10^3/\mu L$	Monocytes cells/μL	Neutrophils $10^3/\mu L$	Eosinophils cells/μL	Basophils cells/μL
group NE									
15.4 ±1.7 (12.3-19.8)	48.0 ±4.3 (40.2-58.4)	10.5 ±0.9 (8.9-12.9)	180.6 ±28.6 (137-242)	7.5 ±2.0 (4.6-12.3)	1.5 ±0.4 (0.8-2.2)	150.8 ±87.5 (30-280)	5.8 ±2.1 (2.5-10.7)	23.6 ±25.8 (10-80)	90.8 ±127.6 (10-380)
group exNE									
14.0 ±2.8 (7.5-18.9)	42.4 ±8.3 (22.4-55.3)	9.8 ±1.8 (5.0-12.6)	175.3 ±61.8 (61-276)	7.7 ±4.2 (0.6-13.9)	1.2 ±0.8 (0.1-3.2)	106.5 ±73.4 (10-260)	6.3 ±4.4 (0.5-12.8)	12.5 ±15.2 (0-60)	56.5 ±54.3 (0-190)
Normal haematology values in one day old foals [34]:									
12.0-16.6	32-46	8.2-11.0	129-409	4.9-11.7	0.7-2.1	70-390	3.4-9.6	0-20	0-30
**Serum biochemistry**									
Creatine kinase IU/L	Total bilirubin mg/dL	Triglycerides mg/dL	Total protein g/dL	Albumin g/dL	Albumin / Globulin	Blood urea nitrogen mg/dL	Creatinine mg/dL	Fibrinogen g/L	Serum amyloid A μg/dL
group NE									
300 ±260 (132-1136)	2.5 ±0.7 (1.3-3.6)	22 ±18 (7-60)	4.2 ±0.3 (3.4-4.5)	3.2 ±0.3 (2.6-3.4)	3.5 ±0.5 (2.7-4.2)	41 ±11 (29-62)	3.6 ±3.1 (1.1-13.1)	1.8 ±0.4 (1.4-2.6)	6 ±7 (1-24)
group exNE									
5511 ±11818 (92-51050)	4.0 ±1.2 (2.0-6.1)	76 ±82 (26-257)	4.5 ±0.4 (3.8-5.1)	3.0 ±0.3 (2.4-3.5)	2.3 ±1.0 (1.3-4.6)	46 ±15 (19-73)	4.0 ±3.3 (0.7-14.8)	2.2 ±0.5 (1.7-3.1)	101 ±170 (1-620)
Normal serum biochemistry values in one day old foals [35,36]:									
40-909	1.3-4.5	30-193	4.3-8.1	2.5-3.6		9-40	1.2-4.3	1-4	0-37

Electrolyte concentrations					
Phosphorus	Calcium	Sodium	Potassium	Chlorine	Magnesium
mg/dL	mg/dL	mg/dL	mg/dL	mg/dL	mg/dL
group NE					
8.7 ±3.8<br>(5.0-14.6)	13.6 ±2.2<br>(9.2-15.7)	143.8 ±2.6<br>(141-149)	5.2 ±0.9<br>(4.3-6.8)	98.6 ±4.2<br>(92.3-102.6)	2.1 ±0.2<br>(1.8-2.5)
group exNE					
5.2 ±2.1<br>(4.0-9.8)	11.9 ±0.6<br>(11.0-12.8)	143.1 ±3.3<br>(139-147)	3.6 ±0.4<br>(3.1-4.0)	99.3 ± 5.6<br>(90.0-105.1)	2.4 ±0.6<br>(1.4-3.9)
Normal electrolyte concentrations in one day old foals [35]:					
3.8-7.4	9.7-13.7	123-159	3.6-5.6	90-114	0.6-4.2

Rapid determinations		
Jugular vein glucose<br>mg/dL	Umbilical vein lactate<br>mmol/L	Jugular vein lactate<br>mmol/L
group NE		
92 ±17<br>(75-109)	3.5 ±0.7<br>(3.0-4.0)	6.4 ±6.6<br>(2.7-22.5)
group exNE		
96 ±64<br>(32-160)	/	9.1 ±7.0<br>(2.5-23.4)
Normal determinations in foals at birth [35,37]:		
67-99	3.2-4.7	2.3-5.0

Arterial blood gas analysis									
pH	Oxygen partial pressure<br>mmHg	Carbon dioxide partial pressure<br>mmHg	Oxygen saturation<br>%	Potassium<br>mmol/L	Sodium<br>mmol/L	Chlorine<br>mmol/L	Anion gap<br>mmol/L	Bicarbonate<br>mmol/L	Acid base excess<br>mmol/L
group NE									
7.37 ±0.04<br>(7.32-7.41)	48.0 ±10.9<br>(34.2-59.9)	43.8 ±4.3<br>(39.4-47.7)	87.9 ±5.1<br>(82.7-93.9)	3.6 ±0.5<br>(3.3-4.4)	140.3 ±1.3<br>(139-142)	99.8 ±4.4<br>(95-104)	19.3 ±7.8<br>(9.8-28.7)	24.2 ±3.6<br>(20.2-28.2)	-0.2 ±4.2<br>(-5.0-4.4)
group exNE									
7.36 ±0.04<br>(7.31-7.41)	66.3 ±17.5<br>(48.5-96.8)	43.7 ±6.9<br>(36.6-54.1)	92.9 ±3.5<br>(87.4-97.2)	2.9 ±0.1<br>(2.6-3.0)	141.4 ±4.3<br>(135-147)	102.4 ±4.3<br>(93-107)	17.9 ±3.7<br>(12.6-22.6)	23.5 ±1.6<br>(21.7-26.4)	-0.9 ±1.9<br>(-3.2-2.5)
Normal arterial blood gas values in foals at birth [38]:									
7.36-7.44	64.2-68.8	46.0-49.4	97-100	3.6-5.6	123-159	90-114	9.5-31.7	24.1-25.9	-0.1-1.9
